# Prevalence, Etiology, and Risk Factors of Tinea Pedis and Tinea Unguium in Tunisia

**DOI:** 10.1155/2017/6835725

**Published:** 2017-08-09

**Authors:** Nourchène Toukabri, Cyrine Dhieb, Dalenda El Euch, Mustapha Rouissi, Mourad Mokni, Najla Sadfi-Zouaoui

**Affiliations:** ^1^Laboratoire de Mycologie, Pathologies et Biomarqueurs, Faculté des Sciences de Tunis, Université Tunis El Manar, 2092 Tunis, Tunisia; ^2^Service de Dermatologie et de Vénéréologie, Hôpital La Rabta, Tunis, Tunisia; ^3^Institut National de la Recherche Agronomique de Tunis, Tunis, Tunisia

## Abstract

**Background:**

Foot mycoses are a frequent disease that represents a public health problem worldwide.

**Objectives:**

This study aims to evaluate the epidemiology of foot mycoses among Tunisian patients, in order to determine the fungal etiological agents and to identify possible risk factors.

**Patients and Methods:**

A prospective study of three hundred and ninety-two patients was undertaken during one year (2013-2014). All subjects were asked to collect demographic data related to the risk factors of foot mycoses. A complete mycological diagnosis was carried out on all patients.

**Results:**

A total of 485 samples were collected; tinea pedis and tinea unguium were confirmed in 88.2% of cases. Dermatophytes were isolated in 70.5% and the most frequent pathogen was* Trichophyton rubrum* (98.1%), followed by yeasts (17.7%) commonly* Candida parapsilosis*. Non-dermatophyte molds (NDMs) were observed in 8.02% cases and* Fusarium* sp. was the frequent genus (29.1%). The main predisposing factors of fungal foot infections were practicing ritual washing (56.6%) and frequentation of communal showers (50.5%).

**Conclusion:**

This is a recent survey of foot mycoses in Tunisia. Epidemiological studies can be useful to eradicate these infections and to provide further measures of hygiene and education.

## 1. Introduction

Fungal infections of the feet including tinea pedis and tinea unguium are very common in the general population [1]. Tinea pedis, generally known as athlete's foot, is divided into three clinical forms such as interdigital, plantar (moccasin foot), and vesiculobullous [2]. Interdigital is the most common clinical manifestation characterized by maceration and fissuring of the skin mainly in the space between the toes. Plantar athlete's foot presents with hyperkeratosic and squamous plaques which cover the soles, heels, and sides of the foot. In inflammatory condition vesicles, pustules and sometimes bullae are present on the sole of the foot [3]. Tinea unguium is classified into four clinical types depending on the mode of penetration of the fungus in the nail plate: distal lateral subungual onychomycosis (DLSO); proximal subungual onychomycosis (PSO); white superficial onychomycosis (WSO); and total dystrophic onychomycosis (TDO) [4].

Because of the prolonged period of treatment and the recurrence of infections, foot mycoses are still considered as a major public health problem affecting quality of life [5]. These fungal infections depends on many factors especially lifestyle and environmental and climatic conditions and can be influenced by individual factors such as age and host defenses [6]. Foot mycoses are mainly caused by dermatophytes, sometimes yeasts, and uncommonly by non-dermatophyte molds (NDMs).

Many epidemiological studies have investigated the variability of the frequency of tinea pedis and tinea unguium in different geographical regions [7–11]. In fact, the practice of epidemiological studies at regular intervals is necessary for monitoring the evolution of foot mycoses over time. To our knowledge, there are few recent studies regarding clinical and mycological features of foot mycoses in Tunisia. The aim of our study was to determine the frequency of foot mycoses, their clinical patterns, predisposing factors, and etiological agents in Tunisian patients.

## 2. Patients and Methods

It was a prospective study that was carried during one year from March 2013 and included all patients referred to the Mycology Unit in the Department of Dermatology and Venereology of La Rabta Universal Hospital in Tunis (Tunisia). Three hundred ninety-two patients were examined to establish the presence of clinical signs of tinea unguium and/or tinea pedis. The questionnaire allowed documentation of potential predisposing factors for foot mycoses, age, sex, diabetes, vascular disease, immunosuppressive drug treatment, psoriasis, fungal infection of the skin, dermatological pathology, associated fingernails onychomycosis, family history of foot mycoses, ritual religious washing, physical activities, used shoes, occlusive shoes, using of publics showers, swimming pools, smoking, walking barefoot, thermal station, pedicure, and the application of henna. Also, the type of tinea pedis (interdigital, hyperkeratosis, and dyshidrosis) and the type of tinea unguium (DLSO, PSO, WSO, or TDO) were documented.

Clinical specimens of skin scrapings and nail clippings were collected in sterile Petri dishes for direct examination and culture. All specimens were submitted to a microscopic examination in Chlorazol noir (Sigma-Aldrich, Germany) solution and inoculated into Sabouraud chloramphenicol dextrose agar with and without cycloheximide (Biorad, France) all in duplicate. The culture was incubated at 27°C and examined after 48–72 h for yeast detection and every four days for at least four weeks for fungal detection.

The identification of filamentous fungi was based on macroscopic and microscopic examination in the Lactophenol Cotton Blue (Sigma-Aldrich, Germany). The identification of yeast was carried out using the API ID 32C system (Biomerieux, France). The mycological examination was considered to be positive if direct microscopic examination and/or culture were positive. In the absence of any dermatophyte or yeast growth, a mold was only considered to be the causal agent of onychomycosis when the culture was repeated on two separate occasions.

Statistical analysis was performed with SPSS software (Statistical Package for Social Scientists version 20.0, SPSS, Inc., Armonk, NY). The Chi-square (*χ*^2^) was used to calculate significant differences in characteristics between patients. Differences with *p* < 0.05 were considered statistically significant.

## 3. Results

A total of 392 patients from various regions in Tunisia were included in this study, 125 males (31.88%) and 267 females (68.11%), with an age range between 3 and 85 years and an average age of 44.7 years. The diagnosis of foot mycosis was confirmed through a mycological diagnosis in 346 (88.26%) cases; the frequency was higher in females (67.05%) compared with males (32.94%) but this prevalence according to the sex was not statistically significant (*p* = 0.217) (Table 1).

**Figure 1:**
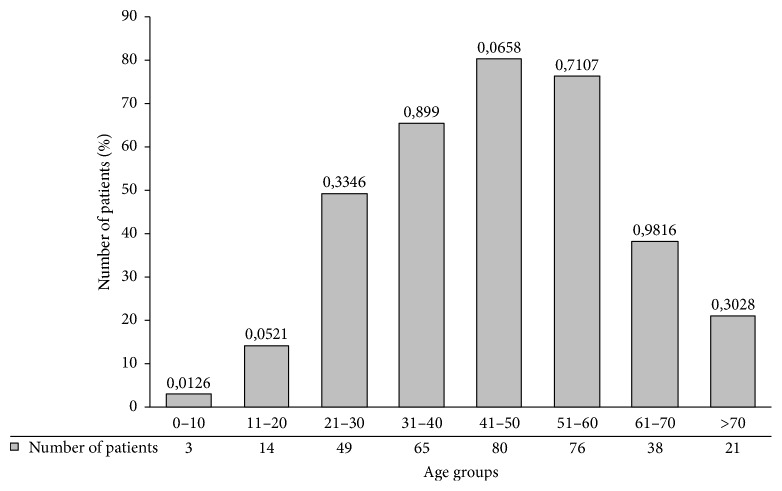
Frequency of foot mycoses according to age groups. Percentage of patients with foot mycoses according to different age groups. *p* value is mentioned under each histogram.

As shown in Figure 1, the frequency of foot mycoses according to the age groups revealed that the patients most commonly infected were between 41 and 50 years (23.1%) followed by those between 51 and 60 years (21.9%) but the differences were not statistically significant (*p* = 0.0658; *p* = 0.71, resp.). However, the prevalence was less frequent in children less than 10 years old (0.8%) and this prevalence was significant (*p* = 0.0126).

Related to the site of infection, we noted that tinea unguium was confirmed in 268 subjects (77.4%) and tinea pedis was confirmed in 78 cases (22.5%). The subtype most frequently observed in tinea pedis was plantar keratoderma in 70 cases (89.7%), followed by interdigital 23 cases (29.4%). 57 cases (73.07%) of the subjects whom presented with tinea pedis have toenail onychomycosis (Table 2).

Clinical patterns of foot mycoses are cited in Table 3; DLSO represent the most common clinical form of tinea unguium (64.3%) followed by TDO (15.6%) and SWO (12.9%). The big toenail was the most infected in 114 cases (35.07%), bilateral nail infection was observed in 55 cases (16.9%), and multiple toes were affected in 103 cases (31.6%). For tinea pedis plantar hyperkeratosis form was observed in 44 cases (62.8%) and plantar dyshidrosis in 26 cases (37.1%). A total of 485 samples were collected from all patients; the direct microscopic examination was positive in 385 specimens (79.3%) showing filaments in 371 cases (96.3%), yeast and pseudomycelium in two cases (0.5%), and both filaments with spores in 12 cases (3.1%).

We have obtained 299 positive cultures (61.6%), including dermatophytes in 211 cases (70.5%), yeasts in 53 (17.7%), NDMs in 24 cases (8.02%), and mixed culture (dermatophytes + yeast) in 11 cases (3.6%). The most frequently isolated dermatophyte was* Trichophyton rubrum* (98.1%), followed by* T. violaceum*,* T. tonsurans*,* T. verrucosum*, and* T. interdigitale* with 0.47% for each species. While* Candida parapsilosis* was the most isolated yeast (60.3%), also* Trichosporon* spp. were isolated (3.7%). The remaining were due to NDMs like* Fusarium* (29.1%),* Penicillium* (25%),* Aspergillus* (20.8%),* Scopulariopsis* (16.6%), and* Scytalidium* (8.3%). In mixed culture,* C. parapsilosis* was most frequently detected with* T. rubrum* (72.7%) (Table 4).

**Figure 2:**
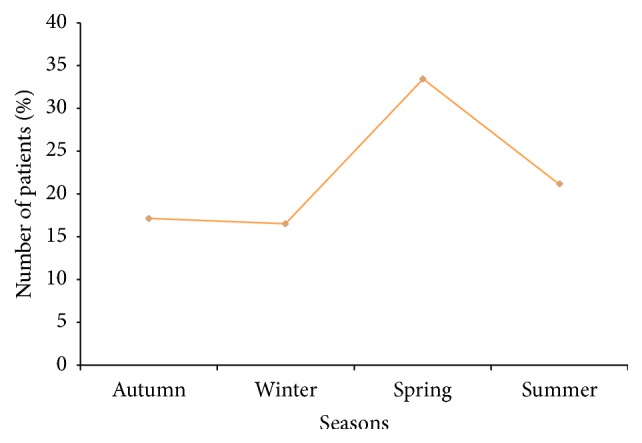
Seasonal evolution of fungal infections of the feet. Percentage of patients consulted during the four seasons.

Since our survey was conducted during one year, we have seen a lower frequency of patients in the winter (16.5%) and most cases were observed in spring (33.4%) and the summer (21.1%) (Figure 2).

Considering the possible risk factors, we noted that the high prevalence was observed in patients who practice ritual ablutions (56.6%) followed by communal shower (50.5%) and family history of foot mycoses (28.6%) but there was no statistically significant association between these factors and foot infection (*p* = 0.41, 0.631, and 0.246, resp.). However, we noted a significant association between foot mycoses and nail trauma (26.5%; *p* = 0.019), wearing used shoes (26.3%; *p* = 0.001), antifungal drugs (25.7%; *p* = 0.013), physical activities (14.7%; *p* = 0.049), occlusive shoes (13.2%; *p* = 0.008), swimming pools (8.09%; *p* = 0.045), attending thermal station (8.3%; *p* = 0.021), pedicure (14.1%; *p* = 0.006), associated fingernails onychomycosis (7.5%; *p* = 0.010), and those taking immunosuppressive drugs (5.4%; *p* = 0.018).

In our study, we did not find a significant association between foot mycoses and diabetes, vascular disease, psoriasis, fungal infection of the skin, dermatological pathology, smoking, obesity, walking barefoot, and the application of henna (*p* > 0.05) (Table 5).

## 4. Discussion

Foot mycosis is the most common superficial infection and represents a major public health problem over the world. Many epidemiological studies have reported the high frequency of this fungal infection, but the prevalence varies with many factors like geographic and demographic parameters and the number of selected population. To our knowledge, the latest epidemiological studies about foot mycoses in Tunisia were established by El Fekih et al. [12] in Tunisia between January and April 2009 and by Dhib et al. [13] during 22 years from 1986 to 2007 in the center of Tunisia (Sousse).

In the present study, the prevalence of tinea unguium and tinea pedis in the population studied were 77.4% and 22.5%, respectively; females were more commonly affected than males which agree with some reports [14–16]. But there was no significant relationship in the occurrence of foot mycoses with respect to the sex and these results are in accordance with Dhib et al. [13]. This may be caused by aesthetics reasons such as repeated aggressive pedicure and manicure, frequent housework, and using detergents that cause nail trauma and generally females consulted more frequently for onychomycosis. However, several studies concluded that males are more infected than females due to the fact that males are more exposed to nail trauma and using occlusive footwear [1, 17].

The frequency of tinea pedis and tinea unguium increased gradually with age; a maximum prevalence was seen in adults aged between 31 and 60 years. These results were confirmed by many studies [10, 12, 18], and this increase may be explained by many conditions such as full-time work activities, frequent nail trauma, reduced nail growth, and inadequate foot care [19]. However, the frequency is less prevalent in the elderly aged between 71 and 80 years and >80 years; this is in agreement with a study reported in Rio Grande do Sul, Brazil [20]. This decreasing frequency can be due to the negligence of old people who do not give importance to nail infections.

Our results also showed that children are rarely infected with foot mycoses; this frequency is in accordance with results observed in school children in Spain [21] and in Turkey [22]. Mycoses infections in children can be due to several factors including the difference in the nail plate, the rapid nail growth, and less exposure to fungal infection than adults.

Tinea pedis is known as the significant reservoir of other dermatophytes in the body and can be a cause of tinea unguium [23]. In the current study, tinea pedis was associated with tinea unguium in more than half of cases (73.07%); this rate was higher than reported in USA [24], in Tokyo [17], and even in another study in Tunisia [12]. This association confirmed the hypothesis that the toenails were infected by toe-webs.

Various clinical patterns of onychomycosis have been reported in the literature. In this work, DLSO was the most frequent clinical form as well as in other studies carried out in Turkey and in Tunisia [25–27]. The most affected toes were the big ones; this observation was expected because of the slow growth of the nail which facilitates the invasion of the pathogenic fungal.

In investigating the causative agents of tinea pedis and tinea unguium, we found that the most common isolated pathogens were dermatophytes [28, 29]; among them,* T. rubrum* was the most common causative agent. These results are similar with other studies [13, 30–32] and are interpreted that* T. rubrum* is a virulent anthropophilic dermatophyte producing arthrospores which have the capacity to persist on the floor surface and on shoes. The second agent responsible for foot mycoses is yeasts, with a high frequency of* C. parapsilosis*. This agrees with the study of El Fekih et al. [12] and can be explained by the fact that* C. parapsilosis* represents a saprophyte yeast of the human skin.

In our results, the anthropophilic* T. violaceum* was isolated from one patient with tinea pedis who had no history of tinea capitis, whereas this species has been classified as the second and the third etiological agent of onychomycosis in few cases as related agent to tinea capitis [26, 33].

Molds are cosmopolitan filamentous fungi; most of them are saprophyte and can be contaminants; however they become opportunistic under unfavorable conditions. In addition to the causative dermatophytes and yeasts, NDMs have been described as etiological agents of foot mycoses. Traditionally, these molds have been considered as secondary pathogens of nails which affect a keratin already degraded and their frequency rates between 1.45 and 17.6% [34]. The primary molds that cause onychomycosis are species belonging to the genus* Scopulariopsis*,* Aspergillus,* and* Fusarium* [35]. In our survey, we found a low incidence of NDMs and the most prevalent species were* Fusarium* sp. (29.1%). This result agreed with the study reported in America showing that* Fusarium* spp. seem to be the most frequent agents [36].

In contrast, a study reported in Italy showed that* Scopulariopsis brevicaulis* was the dominant causative mold [37], although a recent paper from Morocco reports the increasing frequency of* Aspergillus* onychomycosis [38]. In the present work, two cases of onychomycosis were due to* Scytalidium* species. These molds caused lesions similar to those engendered by dermatophytes, called pseudodermatophytes, and widespread in the environment especially in tropical areas [39]. Walking barefoot represents the main risk factor of infections caused by these species because of their telluric reservoir. The presence of NDMs in foot mycoses may be related to many factors that predispose the development of nail infections, such as direct contact with the soil by walking barefoot, wearing open shoes sandals, practicing sports, and trauma of the nail.

Actually onychomycosis caused by NDMs is more prevalent in tropical and subtropical regions with a hot and humid climate making them endemic areas [34, 37].

The contaminated culture can be detected. This may be related to the nonpathogenic nature of the fungi that infects the nail or the presence of a saprophyte mold that inhibits the growth of the pathogenic fungi [11].

In the current study, we note that more cases of fungal infections of the foot were observed in the spring and the summer. The higher frequency during certain seasons can be related to the wearing of occlusive shoes in warm climates, causing heat of the feet which causes maceration and hyperhidrosis, considered as a risk factor of developing foot mycoses. In another way, patients become more aware that the duration of antifungal therapy requires a long period of time and prepare to wear summer shoes because they are more interested in beautiful feet.

Considering the risk factors of foot mycoses, we found a significant association with patients who practice physical activities [32], wear used shoes, or have frequent nail trauma [19] and with patients who receive immunosuppressive therapy [40].

Many other possible risk factors can be related to fungal infections of the foot but our study did not show a significant association; it was found to be most common in persons with family history of fungal infection of the foot [12], having peripheral vascular diseases [41]. Although foot mycoses can be linked to many chronic diseases like diabetes [42], HIV infection [43], and psoriasis [44], this can be explained that people with chronic infections are now more attentive to their health due to awareness raising sessions.

Interestingly, we found a high prevalence of subjects who practice ritual washing. Firstly, this can be explained by the religious custom of ablutions five times every day which can cause maceration of the feet which represents a risk factor of fungal penetration through the stratum corneum of the skin. It also can be related to the spread of fungal species in areas used for washing and in prayer carpets of the mosques; this has been confirmed in other studies [12, 45–47]. Moreover, in the current study, 50.5% of patients with foot mycoses attend communal showers and bathing. This high frequency may be the consequence of the culture and the tradition of Tunisian population to frequent hammams, which are humid and warm locations that are a source of fungal contagion; this also has been found in Algerian population [10, 48].

We observed that some patients have onyxis of both fingers and toes. The association of fingernails onychomycosis can be a risk factor for developing a foot infection that is reported in previous surveys [13, 19]. This association can be related to autoinfection that represents an important source of transmission to another location of the same body [49].

In the present work, 25.7% subjects with foot mycosis have taken antifungal therapy. This finding may be related to the recurrent infection [50] that can be due to various causes which include lack of diagnosis, misidentification of the causative pathogen, and inappropriate choice of antifungal treatment. On the other hand, it can be related to resistant fungal species or the presence of dormant arthroconidia in the nail bed as a reservoir for recurrent infection [51].

## 5. Conclusion

The epidemiological profile of fungal foot infections seems to be related to age, life style, and the presence of comorbidities. Our study shows that the prevalence of these infections is common in the general population of Tunisia, and the frequency is higher than reported in Maghreb, African, and European countries. Our data can be useful to eradicate these infections and provide further measures regarding the personal hygiene and education about prophylaxis in order to reduce the risk factors of tinea pedis and tinea unguium.

## Figures and Tables

**Table 1:** Distribution of foot mycosis according to sex.

	Males		Females		*p* value
	Number	%	Number	%	
Positive	114	91.20	232	86.89	0.217
Negative	11	8.80	35	13.10	
Total	125	31.88	267	68.11	

**Table 2:** Distribution of foot mycoses according to anatomic sites.

Nature of lesion	Site of infection	Number of patients	Direct examination		Culture	
			(+)	(−)	(+)	(−)
Tinea unguium	Nails	268	253	15	201	67
Tinea pedis	Interdigital	3	2	1	3	0
	Plantar	14	13	1	6	8
	Interdigital and plantar	4	4	0	3	1
	Interdigital and nails	5	5	0	5	0
	Plantar and nails	41	41	0	33	8
	Interdigital, plantar, and nails	11	10	1	7	4
Total		346	328	18	258	88

(+): positive; (−): negative.

**Table 3:** Clinical patterns of foot mycoses.

Clinical patterns	Number of cases (%)
*Tinea unguium*	325 (100)
DLSO	209 (64.3)
PSO	23 (7.07)
SWO	42 (12.9)
TDO	51 (15.6)
*Tinea pedis*	78 (100)
Plantar hyperkeratosis	44 (62.8)
Plantar dyshidrosis	26 (37.1)
Interdigital	23 (29.4)

DLSO, distal lateral subungual onychomycosis; PSO, proximal subungual onychomycosis; SWO, superficial white onychomycosis; TDO, total dystrophic onychomycosis.

**Table 4:** Etiological agents responsible for foot mycoses.

Causative agents	Tinea unguium	Tinea pedis	Total (%)
*Dermatophytes*	*165 *	*46 *	*211 (70.5)*
*T. rubrum*	163	44	207 (98.1)
*T. violaceum*	—	1	1 (0.47)
*T. tonsurans*	1	—	1 (0.47)
*T. verrucosum*	—	1	1 (0.47)
*T. interdigitale*	1	—	1 (0.47)
*Yeasts*	*53*	*—*	* 53 (17.7)*
*C. parapsilosis*	32	—	32 (60.3)
*C. tropicalis*	1	—	1 (1.8)
*C. metapsilosis*	2	—	2 (3.7)
*C. famata*	2	—	2 (3.7)
*C. lusitaniae*	1	—	1 (1.8)
*C. pelliculosa*	1	—	1 (1.8)
*C. sake*	2	—	2 (3.7)
*C. guilliermondii*	5	—	5 (9.4)
*Trichosporon asahii*	1	—	1 (1.8)
*Trichosporon mucoides*	1	—	1 (1.8)
*Rhodotorula*	1	—	1 (1.8)
Other* Candida *sp.	4	—	4 (7.5)
*NDMs*	*24*	*—*	* 24 (8.02)*
*Aspergillus *sp.	5	—	5 (20.8)
*Fusarium *sp.	7	—	7 (29.1)
*Scopulariopsis brevicaulis*	4	—	4 (16.6)
*Penicillium *sp.	6	—	6 (25)
*Scytalidium *	2	—	2 (8.3)
*Mixed culture*	*10*	*1*	* 11 (3.6)*
*T. rubrum + C. parapsilosis*	7	1	8 (72.7)
*T. rubrum + C. metapsilosis*	1	—	1 (9.09)
*T. rubrum + Trichosporon *sp.	1	—	1 (9.09)
*T. rubrum + Rhodotorula*	1	—	1 (9.09)

C., *Candida*; T., *Trichophyton*; NDMs, non-dermatophyte molds.

**Table 5:** Possible risk factors associated with foot mycosis based on the questionnaire.

Risk factors	Patients with foot mycosis		*p* value
	Number	%	
*Chronic diseases*			
*Diabetes history*			
Present	41	11.8	0.815
Absent	305	88.1	
*Peripheral vascular disease*			
Present	76	21.9	0.293
Absent	270	78.03	
*Immunosuppressive drugs*			
Present	19	5.4	0.018
Absent	327	94.5	
*Skin disorders*			
*Psoriasis*			
Present	6	1.7	0,368
Absent	340	98.2	
*Fungal infection of the skin*			
Present	7	2.02	0,330
Absent	339	97.9	
*Dermatological pathology*			
Present	11	3.1	0.220
Absent	335	96.8	
*Associated fingernails onychomycosis*			
Present	26	7.5	0.010
Absent	320	92.4	
*Life style*			
*Family history of foot mycosis*			
Present	99	28.6	0.244
Absent	247	71.3	
*Ritual washing*			
Present	196	56.6	0.410
Absent	150	43.3	
*Physical activities*			
Present	51	14.7	0.049
Absent	295	85.2	
*Wearing used shoes*			
Present	91	26.3	0.001
Absent	255	73.6	
*Occlusive shoes*			
Present	46	13.2	0,008
Absent	300	86.8	
*Nail trauma*			
Present	92	26.5	0.019
Absent	254	73.4	
*Swimming pools*			
Present	28	8.09	0,045
Absent	318	91.9	
*Communal shower*			
Present	175	50.5	0.631
Absent	171	49.4	
*Smoking*			
Present	13	3.7	0,181
Absent	333	96.2	
*Obesity*			
Present	8	2.3	0.297
Absent	338	97.6	
*Walking barefoot*			
Present	34	9.82	0,524
Absent	312	90.1	
*Thermal station*			
Present	29	8.3	0.021
Absent	317	91.6	
*Pedicure*			
Present	49	14.1	0,006
Absent	297	85.9	
*Application of henna*			
Present	6	1.73	0.832
Absent	340	98.2	
*Antifungal therapy*			
Present	89	25.7	0.013
Absent	257	74.2	
